# Carbohydrates and exercise performance in non-fasted athletes: A systematic review of studies mimicking real-life

**DOI:** 10.1186/1475-2891-12-16

**Published:** 2013-01-28

**Authors:** Paolo C Colombani, Christof Mannhart, Samuel Mettler

**Affiliations:** 1 SwissFIR Consumer Behavior, ETH Zurich, CH-8092, Zurich, Switzerland; 2 Swiss Federal Institute of Sport Magglingen (SFISM), CH-2532, Magglingen, Switzerland; 3 consulting mannhart, CH-8633, Wolfhausen, Switzerland; 4 Institute of Food, Nutrition and Health, ETH Zurich, CH-8092, Zurich, Switzerland

**Keywords:** Carbohydrates, Performance, Sport, Athlete, Exercise, Ergogenic, Time trial, Athletic performance, Dietary supplement

## Abstract

There is a consensus claiming an ergogenic effect of carbohydrates ingested in the proximity of or during a performance bout. However, in performance studies, the protocols that are used are often highly standardized (e.g. fasted subjects, constant exercise intensity with time-to-exhaustion tests), and do not necessarily reflect competitive real-life situations. Therefore, we aimed at systematically summarizing all studies with a setting mimicking the situation of a real-life competition (e.g., subjects exercising in the postprandial state and with time-trial-like performance tests such as fixed distance or fixed time tests). We performed a PubMed search by using a selection of search terms covering inclusion criteria for sport, athletes, carbohydrates, and fluids, and exclusion criteria for diseases and animals. This search yielded 16,658 articles and the abstract of 16,508 articles contained sufficient information to identify the study as non-eligible for this review. The screening of the full text of the remaining 150 articles yielded 17 articles that were included in this review. These articles described 22 carbohydrate interventions covering test durations from 26 to 241 min (mostly cycling). We observed no performance improvement with half of the carbohydrate interventions, while the other half of the interventions had significant improvement between 1% and 13% (improvement with one of five interventions lasting up to 68 min and with 10 of 17 interventions lasting between 70 and 241 min). Thus, when considering only studies with a setting mimicking real-life competition, there is a mixed general picture about the ergogenic effect of carbohydrates ingested in the proximity of or during a performance bout with an unlikely effect with bouts up to perhaps 70 min and a possible but not compelling ergogenic effect with performance durations longer than about 70 min.

## Introduction

Carbohydrates are one of the two main fuels for sport activities, and their relevance for optimal sport performance is undisputed among experts [1]. Athletes not only ingest carbohydrates as general contributors to their daily energy need but also specifically as ergogenic agents in a more time-specific way, such as during a sport event or in the days preceding it. This potential ergogenic effect of carbohydrates has been the subject of numerous investigations, and a series of reviews have summarized their outcome (e.g., [1,2]). In general, there is a consensus claiming an ergogenic effect of carbohydrates ingested just before or during a performance bout.

One of the fundaments of science is the continuing questioning of current theories in order to corroborate them or, in the case of contradicting new evidence, to challenge them. In the case of the ergogenic effect of carbohydrates ingested near to a performance bout, one could question if the study designs used to investigate this effect were suitable for extrapolating their outcomes to a real-life situation, particularly as a standardized, controlled laboratory setting can be quite different from the conditions of a real-life situation.

Subjects often fast overnight in performance studies. The reason for this is probably that the metabolism in fasted subjects is in a more balanced state, which might be more easily reproduced than a postprandial state. However, the recommendation to athletes is not to compete in a fasted state because of potentially reduced liver glycogen stores and a subsequent negative effect on performance [3]. While this concern might be unsubstantiated (overnight-fasted well-trained subjects can have more than twofold higher liver glycogen levels compared to overnight-fasted non-athletes, ca. 130 g [4] vs. ca. 50 g [5], respectively), athletes almost intuitively do not compete in a fasted state. Further, a test mode assessing how long a subject can exercise at a given intensity is common in “performance” studies (e.g., time-to-exhaustion tests). This is also does not reflect the real-life situation as usually a sporting event, at least in elite sports, requires performing either as fast as possible for a given distance (e.g., races) or as well as possible within a given time (e.g., team sports). See Currell and Jeukendrup for a discussion on the different types of performance tests [6].

In two recent meta-analyses, the ergogenic effect of carbohydrates ingested during endurance sport activities was investigated with the inclusion of fasted subjects [7,8]. In our study, we also aimed at systematically reviewing the influence of carbohydrate intake on performance, but as we wanted to focus on the real-life applicability of the study outcome, we defined a priori to exclude studies with subjects who were fasted and in which the performance test was of a time-to-exhaustion character. However, it was not the focus of this review to discuss why carbohydrate ingestion in the proximity of a performance bout may or may not help athletes.

## Methods

We defined the protocol for conducting the systematic search before commencing the data search and we did not modify it thereafter. One of us (PCC) then screened the PubMed for studies with a carbohydrate intake during the days prior to a sport performance, i.e., a carboloading regime, and studies with a carbohydrate supply immediately before or during a performance bout.

We originally planned to conduct a series of meta-analyses but abandoned this idea when it became clear that the number of studies fulfilling our inclusion criteria was small and the study designs were too heterogeneous for a meaningful grouping.

### Data sources and search strategy

The PubMed was searched up to September 3, 2011 with the following keyword combination: (Exercise OR Sport OR Athlete OR Athletes) AND (Hydration OR Water OR Fluid OR Drink OR Drinks OR Beverage OR Beverages OR Glycogen OR Loading OR Carbo OR Carbohydrate OR Carbohydrates OR Glucose OR Fructose OR Maltodextrin) NOT (Mice OR Mouse OR Pig OR Pigs OR Rat OR Rats OR Horse OR Horses OR Fish OR Dog OR Dogs OR Patient OR Patients OR Disease OR Diseases OR Diabetes OR Obesity OR Obese OR “Cord injury” OR Wheelchair). The plural of several terms was included as we realized that using only the singular of that term yielded a different number of hits (although using the singular of a term should actually also identify abstracts including only the plural of a term). We discarded articles with sufficient information in the abstract that clearly identified the study as ineligible, without consulting the full-text of the article. For the remaining articles, we checked the full-text for the information needed to evaluate the study. We additionally consulted the list of articles judged as eligible for the two meta-analyses mentioned above [7,8].

### Inclusion criteria

We included studies with a randomized, crossover, placebo-controlled, and if possible blinded study design. Blinding was not feasible as an absolute criterion, as sometimes the intervention could not be fully masked (e.g., carboloading vs. no carboloading). Additional criteria were a mean age of the subjects between 18 and 40 years (both genders allowed), a reported VO_2max_ ≥ 50 mL/kg/min body mass, and a performance test in the postprandial state (2 to 4 h after ingesting last meal). The performance test had to be either of a time trial (TT) character, i.e., a fixed distance, fixed time, or fixed amount of work, or a submaximal exercise followed by a TT (S+TT). Further, for studies with a carbohydrate intake immediately prior to and/or during the exercise, we included only studies with provision of any type of carbohydrates, electrolytes, and water, but no further components (e.g., caffeine, choline, protein, amino acids, and fatty acids). We excluded studies with time-to-exhaustion tests assessing the exercise capacity and discarded all studies without sufficient methodological information to enable a check of the inclusion criteria.

### Grouping of interventions

We combined the interventions according to sport type and physiological impact of the activity (in particular with respect to endogenous energy delivery). The three grouping factors were the test mode (cycling, running, or other), a similar test duration (up to 60 min, 61 to 90 min, 91 to 120 min, 121+ min), and a comparable carbohydrate intervention (carboloading vs. no carboloading, ingestion of carbohydrate containing drinks vs. drinks containing no carbohydrates).

### Performance

The outcome was the overall performance during the TT or the TT part in the S+TT interventions as time needed to cover the fixed distance, distance covered within a fixed time, or power accomplished within a fixed time.

## Results

### Eligible studies and interventions

**Figure 1:**
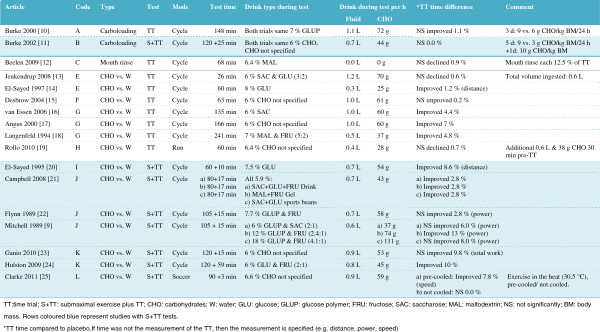
Intervention type, performance bout and outcome of eligible studies.

The PubMed search yielded 16,658 articles and the abstract of 16,508 articles contained sufficient information to identify the corresponding study as not eligible. The full text of the remaining 150 articles allowed the identification of 16 articles that fulfilled the inclusion criteria. We identified one additional eligible article [9] through a scan of the reference lists of both meta-analyses of the similar topic [7,8]. Overall, the 17 articles comprised 22 interventions (14 articles with one intervention, one article with two interventions, and two articles with three interventions, Figure 1).

### Type of studies

Two studies were carboloading interventions; in one study the drink was not ingested (mouth-rinse study), and in the remainder 19 interventions, the effect of a carbohydrate-containing drink was compared to a non-carbohydrate placebo. In four of these 19 interventions, the carbohydrate type was not specified (only the total amount of carbohydrates was reported), while in the other 15 interventions glucose, glucose polymers, maltodextrin, fructose, and/or saccharose were used as carbohydrate sources with a concentration ranging between 5.9% and 18% (mostly between 6% and 7%). Cycling was the exercise mode used with two exceptions: one study with a soccer-specific mode and one study with a running exercise mode. The performance test was a TT with nine interventions (test duration 26 to 241 min) and an S+TT with 13 interventions (test duration 60+10 min to 120+25 min). Overall, a maximum of three studies had a comparable design (same code in Figure 1).

### Characteristics of the subjects

The subjects were men with one exception where both men and women served as subjects. Their mean age ranged from 19 to 35 years and their mean VO_2max_ ranged from 50 to 69 mL/kg body mass/min (Table 1).

**Table 1:** Average characteristics of the subjects of the eligible studies

**Article**	**Number and gender**	**Age[yr]**	**Body Mass Index [kg/m**^**2**^**]**	**VO**_**2**_**max [mL/kg body mass/min]**
Burke 2000 [10]	7 M	28	Not reported	64
Burke 2002 [11]	8 M	28	Not reported	69
Beelen 2009 [12]	14 M	24	21	68^a^
Jeukendrup 2008 [13]	12 M	19	Not reported	66
El-Sayed 1997 [14]	8 M	25	22	67
Desbrow 2004 [15]	9 M	30	Not reported	65
van Essen 2006 [16]	10 M	24	23	63
Angus 2000 [17]	8 M	22	23	65
Langenfeld 1994 [18]	14 M	21	Not reported	56
Rollo 2010 [19]	10 M	34	23	62
El-Sayed 1995 [20]	9 M	24	22	61
Campbell 2008 [21]	8 M / 8 F	35 / 32	24 / 22	59 / 50
Flynn 1989 [22]	7 M	29	23	62
Mitchell 1989 [9]	10 M	24	22	63
Ganio 2010 [23]	14 M	27	23	60
Hulston 2009 [24]	10 M	28	Not reported	62
Clarke 2011 [25]	12 M	25	23	61

M: Males; F: Females.

^a^Calculated from Wmax according to [26].

### Performance outcome

The performance was not significantly different with the following interventions: Both carboloading interventions, the only mouth-rinse intervention, the only running mode intervention, the only cycle TT carbohydrate vs. water intervention up to 60 min, and one of the two cycle TT carbohydrate vs. water interventions between 61 to 90 min (Figure 1). In contrast, a significantly shorter TT time of 4% to 7% was reported with all three cycle TT carbohydrate vs. water interventions 121+ min. For all cycle S+TT carbohydrate vs. water interventions combined, the results were either not significantly different between interventions (four interventions) or favoring the intervention (six interventions, 3% to 13% performance improvement).

Across all interventions (TT and S+TT), the performance was not significantly different compared to a placebo with 11 interventions, while with the remaining 11 interventions the significant performance improvement was between 1% and 13%.

## Discussion

The main finding of this systematic review was that the study design used with the majority of studies investigating the ergogenic effect of carbohydrates ingested in the proximity of or during a performance bout was not suitable for extrapolating the performance outcome to a real-life situation. Further, with half of the eligible studies, either a carboloading regime during the days prior to, or a carbohydrate intake in the proximity of or during a performance test did not significantly improve the TT performance or TT part of an S+TT. It is also noteworthy that no study had subjects with a mean VO_2max_ that would classify them as elite endurance athletes at a high international level (about 70 to 80 mL/kg body mass/min [27]). Studies in which women served as subjects were non existing, except for one study where both genders made up the study population.

### Development of recommendations

Systematic reviews of the scientific literature are considered a scientific and ethical imperative when developing policies and practical recommendations [28]. Nevertheless, systematic reviews are unfortunately not always an integral part of such processes. The probably most prominent negative example was the World Health Organization, where *"Systematic reviews and concise summaries of findings [were] rarely used for developing recommendations. Instead, processes usually [relied] heavily on experts…"*[29]. Relying on experts when developing recommendations is a common procedure and not necessarily problematic. It only becomes a problem if the experts either do not use systematic approaches or do not properly describe the methodology used. Regrettably, the latter often seems to be the case [30]. For example, during the process of performing this review we have encountered articles with missing information on the gender, age, exercise history, or regular training load of the subjects, lacking information on the blinding or randomization of the intervention, and missing information on a pre-exercise meal intake or on the amount of fluids ingested during the performance tests.

### Definition of eligibility criteria

The omission of methodological information in systematic reviews is not the only issue with such analyses. The definition of the inclusion/exclusion criteria is a further crucial step determining the validity of a systematic review [31]. However, no universal set of eligibility criteria exists, as the criteria must be fit for the specific purpose of the review. According to our focus on the real-life portability of the performance outcome, we have used trained subjects exercising in a postprandial state and a performance test similar to a competitive event as main criteria.

These criteria have ultimately led to the exclusion of quite a number of studies, and one could argue that the criteria, therefore, were too restrictive. However, performing in a postprandial compared to a fasted state might indeed be two different things related to the potential ergogenic effect of carbohydrates. In a sub-analysis of the meta-analysis by Temesi et al. [7], the influence of carbohydrate ingestion during endurance performance was investigated according to the fasting state (> 8-h-fasted subjects vs. < 6-h-fasted subjects). The reported effect size of the intervention was similar and significant for both fasting states in the case of S+TT interventions but differed in the case of TT interventions (significant only for the < 6-h-fasted subjects). This result is actually not in line with our review, as we have observed rather mixed results with subjects who were not fasted (although our criteria was 4 to 2 h and not < 6 h). Nevertheless, it indicates that the prandial state might influence a nutritional intervention, and therefore one should consider it with performance studies.

### Attempt in identifying all relevant studies

A second major aspect to consider when conducting a systematic review is the identification of possibly all studies fit for the purpose of the review [31]. A search across multiple databases seems by nature to be more promising in locating more relevant articles than restricting the search to one database only. As we have only searched the PubMed, we may have missed a substantial number of relevant studies, which would have biased our results.

The comparison with the studies identified for the meta-analysis by Temesi et al. [7] in which seven additional scientific databases were searched yielding a total of 41,175 references (compared to 16,658 in our case) and in which less restricting eligibility criteria were applied, led to the identification of only one additional article [9] eligible for our review. The comparison with the meta-analysis by Vandenbogaerde and Hopkins [8], who used Google Scholar for their search, led to the additional identification of the same additional article already identified by the comparison of the meta-analysis by Temesi et al. In contrast, we identified eight articles [10-14,19,21,25] that were not included in the meta-analysis by Temesi et al. [7] and eight articles [10-12,18,19,22,23,25] that were not included in the meta-analysis by Vandenbogaerde and Hopkins [8]. Overall, this indicates that in spite of searching only one database, we likely did not miss a substantial part of published studies on the topic under investigation.

### Concluding remarks

The current consensus indicates that carbohydrates ingested in the proximity of or during a performance bout are ergogenic. However, the application of rigorous criteria to a systematic review, such as excluding fasted subjects and time-to-exhaustion test modes, led to a less convincing picture. We observed no significant performance improvement with most of the performance bouts lasting less than 70 min, and the results with longer performance bouts indicated a significant improvement with 10 of 17 interventions.

The absence of clear evidence is, nevertheless, not clear evidence of an absent effect. This is particularly true for the present review as we discarded many studies because relevant information was missing in the articles. As mentioned above, we encountered studies among other with missing information on age, gender, prandial state, or VO_2max_ of the subjects, missing information on the blinding or randomization of the interventions, or missing information on the drink volume ingested during the intervention. Thus, we cannot exclude that some or even all of these discarded studies would have met the inclusion criteria if only the description were appropriate, and that then the outcome would have been a different one.

The omission of properly describing the methodological part and the frequent use of study designs not allowing an extrapolation of the results to real-life situations are the main reason why it is now difficult to draw a solid conclusion about the potential ergogenic effect of carbohydrates ingested in the proximity of or during a performance bout. Further, it is possible that some of the non-significant studies suffered from low statistical power, particularly studies with just 7 to 8 subjects. Being conservative, however, we can state that with shorter duration events up to perhaps 70 min an ergogenic effect of carbohydrates ingested in the proximity of or during a performance bout is unlikely with trained (but not elite) male athletes in a real-life competition. The picture for longer durations is slightly more in favor than against the current consensus, but it is too heterogeneous for a solid conclusion. Definitely, there is a need for more and more comprehensively described studies to enlighten the current picture.

## Competing interests

All three authors have received honoraria and free products from several companies producing and/or selling carbohydrate-containing sport drinks for diverse purposes (e.g., product development consultations, sponsorships of conferences organized by the authors, talks given to or for the companies). None of the authors holds any patents, stocks or shares, or receives salaries other than mentioned from companies producing and/or selling carbohydrate-containing sport drinks.

## Authors' contributions

All three authors have contributed to the conception and design of the study, and to the analysis and interpretation of the data. PCC acquired the data and drafted the manuscript. CM and SM revised the manuscript and all three authors read and gave final approval of the manuscript version to be published.
